# In-Situ Annealed Ti_3_C_2_T_*x*_ MXene Based All-Solid-State Flexible Zn-Ion Hybrid Micro Supercapacitor Array with Enhanced Stability

**DOI:** 10.1007/s40820-021-00634-2

**Published:** 2021-04-01

**Authors:** La Li, Weijia Liu, Kai Jiang, Di Chen, Fengyu Qu, Guozhen Shen

**Affiliations:** 1 State Key Laboratory for Superlattices and Microstructures, Institute of Semiconductors, Chinese Academy of Sciences and Center of Materials Science and Optoelectronic Engineering, University of Chinese Academy of Sciences, Beijing, 100083 People’s Republic of China; 2 School of Mathematics and Physics, University of Science and Technology, Beijing, Beijing, 100083 People’s Republic of China; 3 Falculty of Hepato-Pancreato-Biliary Surgery, Chinese PLA General Hospital, Institute of Hepatobiliary Surgery of Chinese PLA and Key Laboratory of Digital Hepetobiliary Surgery, Chinese PLA, Beijing, 100853 People’s Republic of China; 4 College of Chemical and Chemistry, Harbin Normal University, Harbin, People’s Republic of China

**Keywords:** Ti_3_C_2_T_*x*_, MXene, Laser writing, Zn-ion hybrid supercapacitor, Flexible energy storage

## Abstract

Zn-ion hybrid supercapacitors (SCs) are considered as promising energy storage owing to their high energy density compared to traditional SCs. How to realize the miniaturization, patterning, and flexibility of the Zn-ion SCs without affecting the electrochemical performances has special meanings for expanding their applications in wearable integrated electronics. Ti_3_C_2_T_*x*_ cathode with outstanding conductivity, unique lamellar structure and good mechanical flexibility has been demonstrated tremendous potential in the design of Zn-ion SCs, but achieving long cycling stability and high rate stability is still big challenges. Here, we proposed a facile laser writing approach to fabricate patterned Ti_3_C_2_T_*x*_-based Zn-ion micro-supercapacitors (MSCs), followed by the in-situ anneal treatment of the assembled MSCs to improve the long-term stability, which exhibits 80% of the capacitance retention even after 50,000 charge/discharge cycles and superior rate stability. The influence of the cathode thickness on the electrochemical performance of the MSCs is also studied. When the thickness reaches 0.851 µm the maximum areal capacitance of 72.02 mF cm^−2^ at scan rate of 10 mV s^−1^, which is 1.77 times higher than that with a thickness of 0.329 µm (35.6 mF cm^−2^). Moreover, the fabricated Ti_3_C_2_T_*x*_ based Zn-ion MSCs have excellent flexibility, a digital timer can be driven by the single device even under bending state, a flexible LED displayer of “TiC” logo also can be easily lighted by the MSC arrays under twisting, crimping, and winding conditions, demonstrating the scalable fabrication and application of the fabricated MSCs in portable electronics.

**Supplementary Information:**

The online version contains supplementary material available at 10.1007/s40820-021-00634-2.

## Highlights

- Flexible Zn-ion hybrid micro-supercapacitors (MSCs) array was fabricated with Ti_3_C_2_T_*x*_ as the cathode via laser direct writing method, which present ultrastability up to 50,000 cycles after in-situ annealed treatment.
- A digital timer driven by the obtained single MSC under bending state, together with a flexible LED displayer of the “TiC” logo lighted by the MSC arrays under twisting, crimping and winding conditions demonstrate the great potential application of the MSCs in integrated wearable electronics.

**Supplementary Information:**

The online version contains supplementary material available at 10.1007/s40820-021-00634-2.

## Graphical abstract

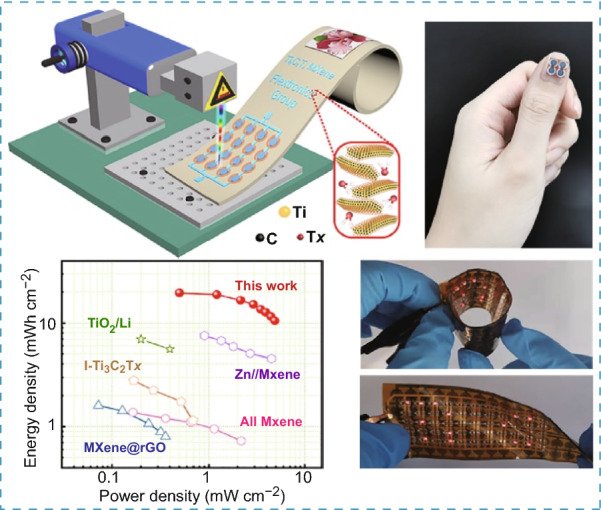

## Introduction

To meet the demands of flexible and wearable devices including various sensors, detectors, transistors, memristors, etc*.*, the matched energy storage must satisfy both the external requirements of miniaturization, patterning, integration, and comfortableness and internal needs of the superior charge storage capability [1–5]. Excluding the harmful fuel cells, capacitive-type electrode-based supercapacitors (SCs) and diffusion-type electrode-based batteries present their own merits and demerits. Alternatively, metal-ions hybrid SCs could exert the strength of high power density, security, and outstanding stability coming from SCs as well as excellent energy density derived from battery [6–8]. Flexible Zn-ion hybrid supercapacitors (SCs) that consist of the flexible substrate, Zn anode, functional cathode as well as gel electrolyte have garnered significant interest owing to their high specific capacity of 823 mAh g^−1^, the low redox potential of − 0.76 V (vs. standard hydrogen electrode), stability, natural abundance, and non-pollution compared to other metal-ion hybrid SCs [9–11]. Functional cathode as the key component affects the electrochemical performance of Zn hybrid SC the most [12–14]. 2D transition-metal carbides and nitrides (MXenes) with the general formula M_n+1_X_n_T_*x*_, where M is an early transition metal, X represents carbon and/or nitrogen, T_*x*_ stands for hydroxyl (–OH), oxygen (–O), or fluorine (–F) termination, n = 1–4 [15], that possesses the features of high conductivity, excellent flexibility, adjustable chemistry, and scalability since the first found in 2011 [16], are widely used in the area of transistors [17], sensors [18, 19], electromagnetic interference shielding [20], catalysis [21], adsorbents [22], particularly in the high-performance energy storage field like photovoltaic cell, metal-ion battery, SCs [23, 24].

Large-scale and efficient manufacturing technology is of great significance to fabricate flexible Zn-ion hybrid SCs and solve the mass production problem. In comparison with the printed process, laser direct writing method of functional materials has been demonstrated the universal adaptability, facility and variable-area patterns with high-resolution, which have no requirement for the solvents, the utilization of binder, additives and the adjustment of the viscosity, surface tension, and wettability of the materials to be processed [25]. Up to now, many high-performance energy storage devices have been developed using laser direct writing technology. For instance, Liu et al*.* [26] provided an active carbon-based flexible planar interdigitated solid-state supercapacitors based on active carbon electrodes (areal specific capacitance up to 34.7 mF cm^−2^ at the current density of 0.1 mA cm^−2^) by employing laser writing methods, but the addition of silver paint current collector complicate the fabrication process. Peng and co-workers [27] reported on an all-Ti_3_C_2_T_*x*_ MXene-based solid-state interdigital micro-supercapacitors (MSCs) via laser cutting technology, high conductive Ti_3_C_2_T_*x*_ MXene with large size serve as current collector deeply simplified manufacturing process. However, Ti_3_C_2_T_*x*_ MXene as cathode material in the design of Zn-ion hybrid SCs suffers from poor cycling performance and rate stability, more efforts should be continuously devoted to solving these problems.

Combing the Ti_3_C_2_T_*x*_ cathode and low-cost laser writing manufacturing routes, we provided in-suit annealed the fabricated all-solid-state flexible Zn-ion hybrid MSCs on the polyimide substrate at 300 °C for 30 min in Ar atmosphere, showing ultrastability up to 50,000 cycles owing to the removal of the surface oxygen-containing group and the formation of the micropores in Ti_3_C_2_T_*x*_ structures. To maximize Ti_3_C_2_T_*x*_ cathode utilization, the optimal thickness of electrodes was discussed in this work. The assembled flexible Zn-ion hybrid MSCs after annealed treatment exhibited the maximum areal capacitance of 72.02 mF cm^**−**2^ at scan rate of 10 mV s^**−1**^ with a thickness of 0.851 µm (662.53 F cm^**−**3^), and provided a power density of 0.50 mW cm^**−**2^ at an area energy density of 0.02 mWh cm^**−**2^. The flexible MSCs with different patterns and series/parallel combinations also were designed. A digital timer driven by the obtained single MSC under bending state, together with a flexible LED displayer of “TiC” logo lighted by the MSC arrays under twisting, crimping and winding conditions proved the superior performance of the fabricated devices, opening up the way for the feasible prototype of scalable high-performance MSCs fabrication and enhancing the application forms of the MSCs devices in integrated wearable electronics.

## Experimental Section

### Material Synthesis

#### Materials

Ti_3_AlC_2_ MAX was purchased from Carbon-Ukraine. Hydrofluoric acid (HF, 40%), hydrochloric acid (HCl, 9 M), lithium chloride (LiCl, 99%), Zinc chloride (ZnCl_2_, 99%) were procured from Aladdin Industrial Inc. Polyvinylalcohol (PVA; MW = 70,000–100,000) was provided by Himedia Laboratories Pvt. Ltd., and polyimide (PI) substrate was supplied by Dupont. Kapton, poly-(dimethylsiloxane) (PDMS) was obtained from SYLGARD 184 Silicone Elastomer Base and Curing Agent. Double distilled water was used for all the experiments. All chemicals were used directly without further purification.

#### ***Mono or Fewer Layer Ti***_***3***_***C***_***2***_***T***_***x***_*** MXene***

The selective etching method was carried out according to our previous work. In the typical process, 1.5 g of Ti_3_AlC_2_ MAX phase was slowly added into a mixture of 13.5 mL deionized (DI) water, 13.5 mL HCl (9 M), and 3 mL HF, and stirred at room temperature for 24 h. The sediment was washed with DI water several times until the pH value reached neutral, then dried at 80 °C for 12 h under vacuum to obtain multi-layer Ti_3_C_2_T_*x*_ MXene. Subsequently, 1 g of multi-layer Ti_3_C_2_T_*x*_ MXene was added into 15 mL DI water containing 1.5 g of LiCl under vigorous stirring at room temperature for 12 h. The Ti_3_C_2_T_*x*_ suspension with large size was washed with DI water several times and collected by centrifugation at 3500 rpm for 5 min assisted by handshaking, while small-sized Ti_3_C_2_T_*x*_ MXene was obtained after sonication treatment.

### Fabrication Process

#### Fabrication of Patterned MSCs

The cleaned PI (Kapton HN, DuPont, 100 µm) substrate was put into plasma cleaner for 5 min to enhance the hydrophility and wettability. Then, the large-sized Ti_3_C_2_T_*x*_ suspension with a concentration of 3 mg mL^−1^ was spread on the PI substrate and dried at 80 °C for 20 min under vacuum. After that, a laser direct writing process (Power, 20 W) was performed to pattern the electrodes. The patterns were cut by four times. The number of device arrays and the electrode structure can be designed by the preset pattern.

#### Fabrication of Patterned MSCs

Zn anode was directly obtained via electrodeposition process. A three-electrode system for electrodeposition that consists of the patterned large-sized Ti_3_C_2_T_*x*_ as the working electrode, a Pt plate as the counter electrode, and Hg/HgO electrode as the reference electrode was employed. The electrolyte aqueous was prepared by adding 0.09 M of Na_2_SO_4_, 0.045 M of ZnSO_4_·7H_2_O and 0.05 M of H_3_BO_3_ to 30 mL DI water. A constant voltage of − 1.5 V for 1 min was applied to prepare Zn film. Afterward, another Ti_3_C_2_T_*x*_ electrode was coated with small-sized Ti_3_C_2_T_*x*_ as active materials several times (labeled as layer count). The fabricated devices were then annealed at 300 °C for 30 min in Ar atmosphere and naturally cooled down to remove surface group and improve the stability. Finally, the devices were encapsulated by the transparent and ultrathin PDMS film after spreading the PVA/ZnCl_2_ gel electrolyte.

#### Preparation of Gel Electrolyte

The PVA/ZnCl_2_ gel electrolyte was prepared by adding 3 g PVA and 6 M ZnCl_2_ in 30 mL DI water with stirring at 98 °C for 30 min until the solution became clear.

### Characterization

The electrochemical performances of the assembled micro Zn-ion supercapacitors were measured by the CHI 760D electrochemical workstations. The surface morphology of the multi and monolayer Ti_3_C_2_T_*x*_ MXene was investigated via the scanning electron microscopy (SEM) system (NANOSEM 650-6700F) and transmission electron microscopy (HRTEM; JEOLJEM-2010HT). The crystallinity of the prepared samples was collected by powder X-ray diffraction (Rigaku D/Max-2550). The surface morphology and thickness of single Ti_3_C_2_T_*x*_ flakes were observed by AFM (Bruker Multimode 8). The thickness of the Ti_3_C_2_T_*x*_ film was conducted by a step profiler (Dektak XT, Bruker, Billerica, MA, USA).

## Results and Discussion

### Material Characterizations

**Fig. 1:**
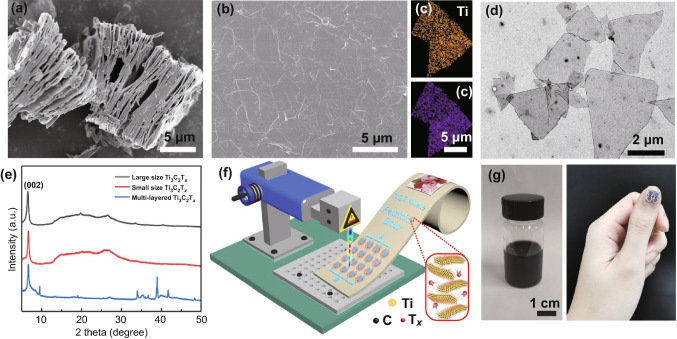
Morphology, crystallinity of the synthesized Ti_3_C_2_T_*x*_ materials, and the fabrication process of the Zn-ion hybrid MSCs array. **a** SEM image of the multi-layer Ti_3_C_2_T_*x*_ MXene. **b** SEM image of the monolayer Ti_3_C_2_T_*x*_ MXene with large size. **c** Elemental mapping images showing the dispersion of Ti and C elements in the Ti_3_C_2_T_*x*_ MXene. **d** TEM image of the sonicated monolayer Ti_3_C_2_T_*x*_ MXene with small size. **e** XRD patterns of the multi and monolayered Ti_3_C_2_T_*x*_ MXenes. **f** Schematic diagram exhibiting the fabrication process of the patterned MSCs array via laser writing technology. **g** Digital photos of the Ti_3_C_2_T_*x*_ supernate and the fabricated micro device array directly attached to the fingernail

The structural and morphological characterizations of the synthesized Ti_3_C_2_T_*x*_ materials are revealed in Fig. 1. Figure 1a shows the SEM image of the multi-layer Ti_3_C_2_T_*x*_ MXene, where the typical accordion-like structures produced by the HCl/HF etching method can be found [28]. The delamination process is then carried out to obtain monolayer or few-layer MXenes flakes using Li^+^ metal cations intercalation, the prepared 2D large-sized Ti_3_C_2_T_*x*_ materials assisted with handshaking can be seen in Fig. 1b. Figure 1c depicts the corresponding mapping images of Ti and C elemental magnified in the selected areal of Fig. 1b. It can be seen from the dot mapping images of the elements that C and Ti uniformly distribute in the 2D Ti_3_C_2_T_*x*_ nanosheets. The large-sized Ti_3_C_2_T_*x*_ nanosheets (lateral size > 5 µm) with high conductivity and fewer defects are an excellent candidate for the current collector, while the small-sized Ti_3_C_2_T_*x*_ flakes with abundant surface defects after the sonication treatment possess better electrochemical performance. Figure 1d exhibits the TEM image of the sonicated Ti_3_C_2_T_*x*_ flakes. Obviously, the Ti_3_C_2_T_*x*_ flakes showed an average lateral size of 1 µm. The atomic force microscope (AFM) image (Fig. S1) was provided to show the thickness of the single Ti_3_C_2_T_*x*_ flakes. Based on Fig. S1, we can see the thickness is about 3.54 nm. The crystallinity and phase composition of the multi-layer, monolayer Ti_3_C_2_T_*x*_ nanoflakes with large and small size is presented in Fig. 1e. The (002) peaks for the monolayer Ti_3_C_2_T_*x*_ MXene are located at ~ 6.7° and shifted to the left compared to multi-layer Ti_3_C_2_T_*x*_ MXene because of the existence of water molecules between MXene flakes [29]. Combined with the morphology and XRD results, it can be concluded that the monolayer Ti_3_C_2_T_*x*_ MXene is successfully produced.

Figure 1f displays the laser writing manufacturing process of the Ti_3_C_2_T_*x*_ MXene-based Zn-ion hybrid MSCs on the flexible PI substrate. The spin-coated large-sized Ti_3_C_2_T_*x*_ current collector was produced according to the designed pattern. Subsequently, the Zn anode was prepared via the electrochemical deposition method. Then, the small-sized Ti_3_C_2_T_*x*_ cathode with various thicknesses was sprayed. Finally, PVA/ZnCl_2_ gel electrolyte was spread on the devices. The digital photo of the Ti_3_C_2_T_*x*_ suspension is displayed in Fig. 1g (left). The MSC arrays (4 in parallel) could be directly attached to the fingernail, exhibiting the wearable features and easy integration in the flexible electronics.

### Device Fabrication

**Fig. 2:**
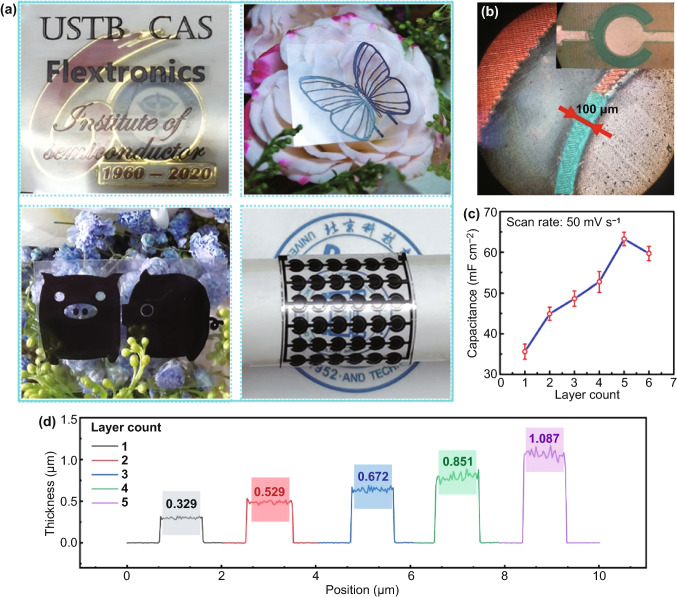
Design of MSC devices with various patterns, electrode structure, and array on flexible PET substrate. **a** Optical images of laser written “USTB, CAS, Flextronics, Institute of semiconductor” words, cartoon MSC, butterfly-shaped Zn-ion MSC, and MSC array. **b** Microphotography of the concentric circular MSC with a 100 µm gap between Zn anode and Ti_3_C_2_T_*x*_ cathode. **c** Capacitance of the Ti_3_C_2_T_*x*_ cathode with increasing layers in Zn-ion MSC. **d** Thickness of the Ti_3_C_2_T_*x*_ cathode related to different layer counts

Figure 2a depicts the digital image of various fine-patterned MXene electrodes, such as “USTB” “CAS” “Flextronics” “Institute of semiconductor” words on a transparent PET substrate (size 3 × 3 cm^2^), showing the universal adaptability of the laser writing method. A cartoon MSC was also fabricated to prove the quick and easy fabrication process. We also prepared a Zn-ion MSC with butterfly-shaped, one wing was deposited with Zn, which demonstrates the possibility to design the MSC based on the wearable electric apparatus. The energy density can be controlled by the series/parallel-multiple connection, as shown in the bottom right corner of Fig. 2a. The optical microscope image of the planar concentric circular Zn-ion hybrid MSC is displayed in Fig. 2b. A legible track with a width of 100 μm divides the Zn anode and Ti_3_C_2_T_*x*_ cathode. To improve the Ti_3_C_2_T_*x*_ cathode utilization, the optimal thickness of electrodes was discussed in Fig. 2c. Obviously, as the deposited Ti_3_C_2_T_*x*_ layers increase, the areal capacitance of the fabricated Zn-ion hybrid MSC is increased first, and then decreased when the layer count reaches 6. The thickness of the Ti_3_C_2_T_*x*_ cathode with increased layer count was then measured by the step profiler, as displayed in Fig. 2d. The optimum average thickness is about 1.087 µm, which is used as the standard in the following MSC devices fabrication process.

### Electrochemical Performances Analysis

**Fig. 3:**
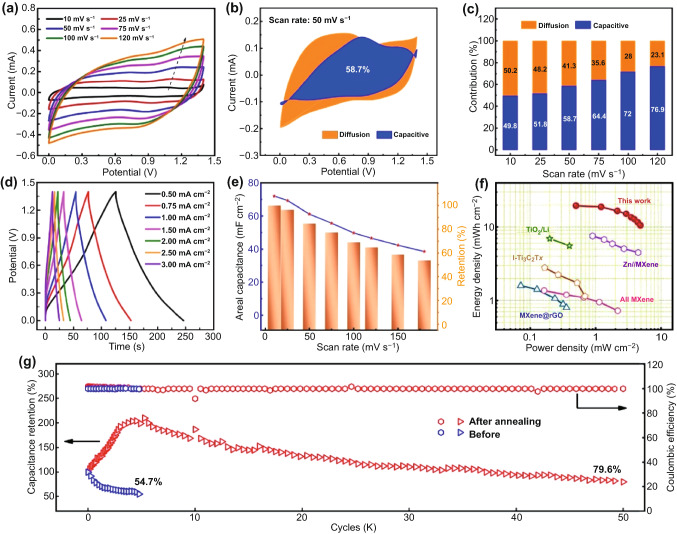
Electrochemical performances of the Ti_3_C_2_T_*x*_-based concentric circular Zn-ion MSC. **a** Cyclic voltammetry (CV) profiles of the fabricated MSCs without annealing treatment at different scan rates varying from 10 to 120 mV s^−1^. **b** Capacitive and diffusion current of the in-situ annealed MSCs at a scan rate of 50 mV s^−1^. **c** Capacitive and diffusion contribution ratio of the in-situ annealed MSCs at different scan rates. **d** Galvanostatic charge–discharge (GCD) curves of the in-situ annealed MSCs at different current density (0.5–3.0 mA cm^−2^). **e** Variation of areal capacitance with various scan rates. **f** Ragone plot of areal energy density vs. power density for the fabricated in-situ annealed MSC in comparison with the reported Zn-ion devices. **g** Cycling stability of the fabricated MSC

The electrochemical performances of the fabricated Ti_3_C_2_T_*x*_-based concentric circular Zn-ion hybrid MSC were systematically gathered, as shown in Fig. 3. Figure 3a displays the CV curves of the fabricated MSCs without annealing treatment at the scan rates ranging from 10 to 120 mV s^−1^. The CV curves exhibited a quasi-rectangular shape in a voltage window of 1.4 V at low scan rates and the small humps during both the cathodic and anodic sweeps should be contributed to the insertion and ejection of Zn-ion. The GCD curves at various current densities rates from 0.5 to 3 mA cm^−2^ are exhibited in Fig. S2a. The triangular shape of the GCD curve is typical of capacitive and reversible electric double layer capacitive behavior. High areal specific capacitance of 112 mF cm^−2^ was obtained at a scan rate of 10 mV s^−1^ (Fig. S2b). The rate capability of the fabricated Zn-ion hybrid MSCs with gradually increased current is displayed in Fig. S2c. No noticeable decrease after 40 times of continuous cycling at varied current densities ranging from 0.5 to 3 mA cm^−2^ can be seen. However, after 50,000 charge–discharge cycles, only ~ 54.7% value of its initial capacitance remained for the fabricated devices. Hence, the MSC was annealed at 300 °C for 30 min in Ar atmosphere to remove the surface group and improve the cycling stability.

The CV profiles of the annealed Ti_3_C_2_T_*x*_-based Zn-ion hybrid MSCs are revealed in Fig. S3a, which are quite similar to those of the MSCs without treatment, demonstrating the thermal treatment has no damage to the structure of the Ti_3_C_2_T_*x*_ cathode. To explore the hybrid kinetics of the annealed hybrid MSCs, the separated capacitive current and diffusion current is displayed in Fig. 3b [30]. 58.7% of the total current came from the capacitive-controlled process at a scan rate of 50 mV s^−1^, which increased to 76.9% when the scan rate increases to 120 mV s^−1^ (Fig. 3c). The GCD curves of the annealed hybrid MSCs in Fig. 3d exhibit the same triangular shape. The compared areal capacitance of the MSCs with or without annealing treatment (Fig. S3b) shows that, with the scan rate increased, the capacitance of the annealed hybrid MSCs goes near to that of the MSCs without treatment. Another important reason that caused the capacitance reduction for the annealed hybrid MSCs is the lack of the cycling activation process, which will be discussed later. The highest capacitances based on the CV curves were calculated to be 72.02 mF cm^−2^ at a scan rate of 10 mV s^−1^, and 662.53 F cm^−3^, respectively, as shown in Figs. 3e and S3c. The comparison of energy and power densities of our devices and other types of SCs was plotted in the Ragone plot in Fig. 3f. Our devices delivered the largest areal energy density of 0.02 mWh cm^−2^ at an area power density of 0.50 mW cm^−2^, the largest volume energy density volume of 0.18 mWh cm^−3^ at a volume power density of 0.024.63 mWh cm^−3^ (Fig. S3d), which is much higher that of TiO_2_//Li hybrid SCs (6.94 µWh cm^−2^, 0.20 mW cm^−2^) [31], Zn//MXene hybrid SCs (7.53 µWh cm^−2^, 0.90 mW cm^−2^) [32], I-Ti_3_C_2_T_*x*_ SCs (2.8 µWh cm^−2^, 0.17 mW cm^−2^) [33], Ti_3_C_2_T_*x*_ MSCs (1.37 µWh cm^−2^, 0.16 mW cm^−2^) [27], and MXene@rGO MSCs (1.6 µWh cm^−2^, 0.07 mW cm^−2^) [34]. The cycling stability (Fig. 3g) was also measured to highlight the ultrastability of the annealed devices, which remains ~ 80% value of its initial capacitance after 50,000 galvanostatic charge/discharge cycles, the active process can be clearly seen from the figures, which is caused by the removal of the surface oxygen-containing functional group and the formation of the micropores in Ti_3_C_2_T_*x*_ structures. Moreover, electrochemical impedance spectra (EIS) of the annealed devices (Fig. S4) show a low resistance of 47 Ω owing to the high conductivity of the Ti_3_C_2_T_*x*_ cathode.

### Investigation of the Charge Storage Mechanism

**Fig. 4:**
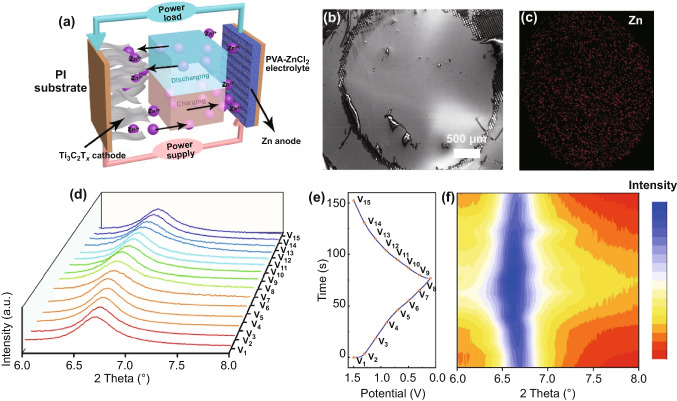
Mechanism study of the Ti_3_C_2_T_*x*_-based concentric circular Zn-ion MSC. **a** Schematic illustration of the Ti_3_C_2_T_*x*_-based Zn-ion MSC during the discharging/charging process. **b** SEM image of the Ti_3_C_2_T_*x*_ cathode after charging. **c** The corresponding Zn element dispersion in the Ti_3_C_2_T_*x*_ cathode. **d–f** Ex-situ XRD patterns of the Ti_3_C_2_T_*x*_ cathode

To further understand the charge storage mechanism of the Ti_3_C_2_T_*x*_-based Zn-ion hybrid MSC, we provide the schematic illustration of the mechanism, as displayed in Fig. 4a. During the discharge process, Zn transforms to Zn^2+^ and moves from anode to cathode, then intercalates into the Ti_3_C_2_T_*x*_ layers or adsorbs on the surface of the Ti_3_C_2_T_*x*_ cathode. When the MSC device charges, the procedure is the inverse of the above process. This mechanism is also demonstrated by the in/ex-situ SEM and XRD analysis. Figures 4b, c and S5 show the SEM image of the Ti_3_C_2_T_*x*_ cathode and the corresponding homogeneous Zn, C, Ti element dispersion in the Ti_3_C_2_T_*x*_ cathode after charging, proving the insertion/adsorption of the Zn^2+^ in the Ti_3_C_2_T_*x*_ cathode. The ex-situ XRD analysis (Fig. 4d–f) is then presented to further explain the charge storage mechanism. From the XRD patterns, we can see when the voltage changes from 1.4 to 0 V, the characteristic peak (002) of Ti_3_C_2_T_*x*_ cathode at 6.7° gradually shifts to the left, revealing the interlayer space becomes wider, which means Zn^2+^ intercalates into the interlayer of the Ti_3_C_2_T_*x*_ cathode. Instead when the device charges from 0 to 1.5 V, the characteristic peak gradually shifts to the initial position. This reversible charge/discharge process ensures the outstanding cycling performance of the fabricated devices.

### Flexibility and Application of the Fabricated MSC Arrays

**Fig. 5:**
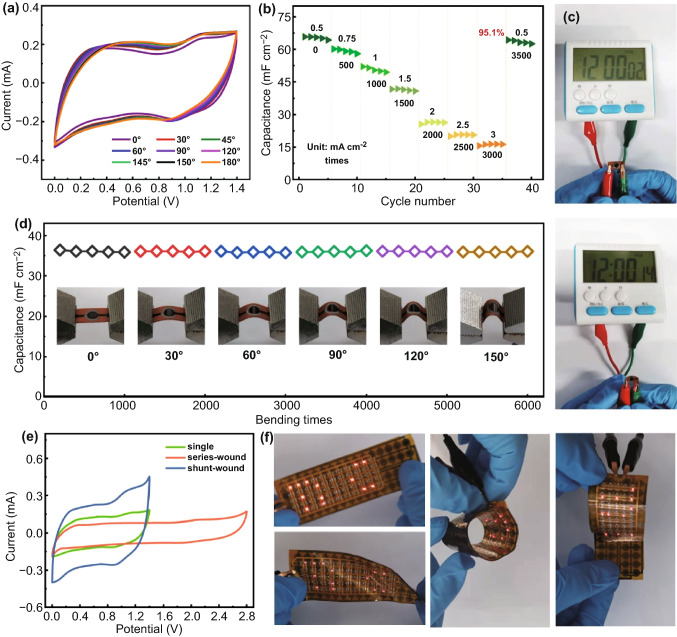
Flexibility and rate stability of the fabricated Ti_3_C_2_T_*x*_-based Zn-ion MSC. **a** CV curves of the designed MSC under the different bending state. **b** Areal capacitance of the MSC at different current densities and bending times. **c** Photographs of the digital timer driven by the single Ti_3_C_2_T_*x*_-based Zn-ion MSC under a flat and bending state. **d** Areal capacitance change of the fabricated MSC under different bending states for several bending cycles. **e** CV curves of the as-prepared two MSCs in series and parallel. **f** Digital images of the Ti_3_C_2_T_*x*_-based Zn-ion MSC array powering a flexible LED array of the “TiC” logo under various types of deformations like twisting, crimping, and winding

Owing to the utilization of the flexible PI substrate and all-solid-state PVA/ZnCl_2_ gel electrolyte, the assembled Ti_3_C_2_T_x_-based Zn-ion MSC presents the superior flexibility, as shown in Fig. 5a. The CV curves of the MSC devices reveal nearly constant shape when the bending state varies from 0° to 180°, showing excellent mechanical stability. The rate stability of the devices after bending for 500 times at each current density in Fig. 5b shows 95.1% of the initial value, which possesses great potential in the fast-charged Zn-ion hybrid MSCs. Figure 5c displays the optical photographs of the digital timer powered by a single Ti_3_C_2_T_x_-based Zn-ion hybrid MSC under a flat and bending state. The real-time video (Movie S1) demonstrates the digital timer can work continuously for more than 5 min under repetitive bending-flat state, showing an intuitive application in wearable devices and providing new design ideas for the flexible devices. The stability of the Ti_3_C_2_T_x_-based Zn-ion hybrid MSC under different bending times is also measured, as shown in Fig. 5d. The nearly invariable areal capacitance after bending for 1000 times under each bending state reveals non-reducing capacitance, suggesting its excellent flexibility. The inset shows the digital photos of the fabricated MSCs fixed on the 1D platform from the original state to different bending states. To enlarge the output potential and energy density of the fabricated MSCs, two MSCs connected in series and parallel were designed, respectively, as shown in Fig. 5e, which showed double output voltage or current with the same scan rate. Figure 5f shows the flexible LED array of the “TiC” logo that was lighted by the MSCs array. It can be seen that the LED array even can be powered under various types of deformations like twisting, crimping, and winding. The corresponding real-time video is provided in Movies S2 and S3, providing robust support for the flexible displays or functional wearable and portable electronics.

## Conclusions

In summary, a high-performance flexible Zn-ion hybrid MSC constructed by a Ti_3_C_2_T_*x*_ cathode and low-cost laser writing manufacturing routes has been successfully fabricated. The cycling stability of the prepared MSCs was greatly improved because of the in-suit annealed treatment at 300 °C for 30 min in Ar atmosphere, which showed robust cycling stability with 80% of the capacitance retention even after 50,000 charge/discharge cycles. The obtained flexible Zn-ion hybrid MSCs after annealed treatment exhibited a high areal capacitance of 72.02 mF cm^**−**2^ at a scan rate of 10 mV s^**−1**^ with a thickness of 0.851 µm, (662.53 F cm^**−**3^), and provided a power density of 0.50 mW cm^**−**2^ at an area energy density of 0.02 mWh cm^**−**2^. A digital timer can be driven by the single MSC even under bending state, a flexible LED displayer of the “TiC” logo was lighted by the prepared MSC arrays under different deformations, which demonstrated the high-performance of the fabricated MSCs and provided great potential applications in the next-generation portable electronics and devices.

## Supplementary Information

Below is the link to the electronic supplementary material.

Supplementary file1 (PDF 526 KB)
