# Supplementary material for: In-Situ Annealed Ti3C2Tx MXene Based All-Solid-State Flexible Zn-Ion Hybrid Micro Supercapacitor Array with Enhanced Stability
Source: Nanomicro Lett. 2021 Apr 1;13:100. doi: 10.1007/s40820-021-00634-2 (PMC8017055; doi:10.1007/s40820-021-00634-2)
Supplement: Supplementary file 1 — Supplementary file1 (PDF 526 KB) [file 40820_2021_634_MOESM1_ESM.pdf]

Supporting Information for

In-situ Annealed $Ti_3C_2T_x$ MXene Based All-Solid-State Flexible Zn-Ion Hybrid Micro Supercapacitor Array with Enhanced Stability

La Li[1, +], Weijia Liu[2, +], Kai Jiang[3], Di Chen[2, *], Fengyu Qu[4], Guozhen Shen[1, *]

[1]State Key Laboratory for Superlattices and Microstructures, Institute of Semiconductors, Chinese Academy of Sciences & Center of Materials Science and Optoelectronic Engineering, University of Chinese Academy of Sciences, Beijing 100083, P. R. China

[2]School of Mathematics and Physics, University of Science and Technology Beijing, Beijing 100083, P. R. China

[3]Falculty of Hepato-Pancreato-Biliary Surgery, Chinese PLA General Hospital, Institute of Hepatobiliary Surgery of Chinese PLA & Key Laboratory of Digital Hepetobiliary Surgery, Chinese PLA, Beijing 100853, P. R. China

[4]College of Chemical and Chemistry, Harbin Normal University, Harbin, P. R. China

[+]L. Li and W. Liu contributed equally to this work

*Corresponding author. E-mail: chendi@ustb.edu.cn (Di Chen), gzshen@semi.ac.cn (Guozhen Shen)

Supplementary Tables and Figures

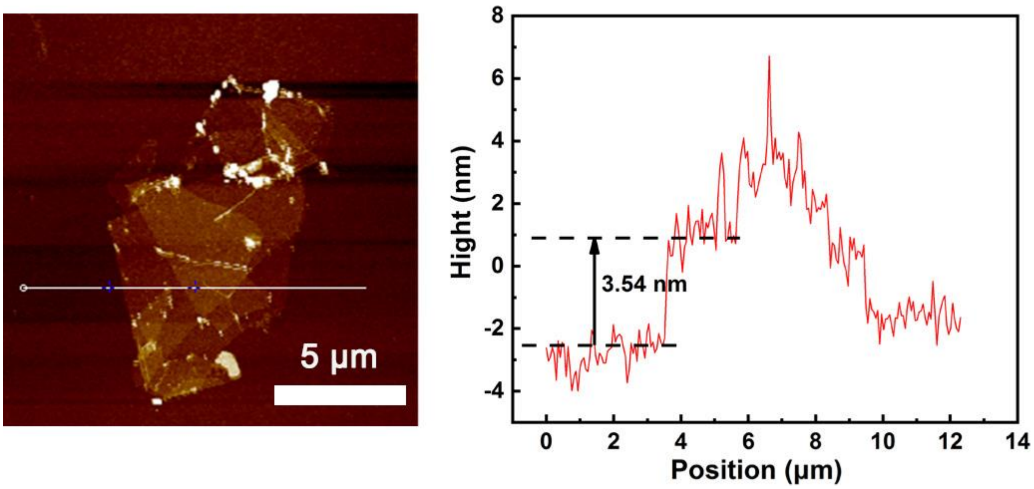

Fig. S1 AFM images of the $Ti_3C_2T_x$ flakes

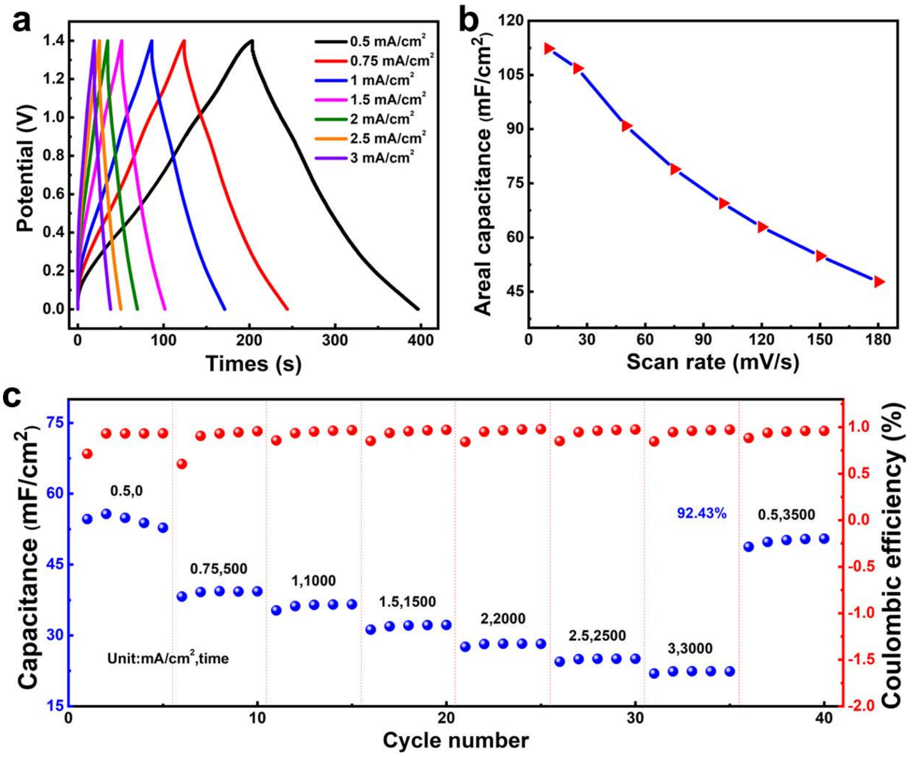

Fig. S2 a Galvanostatic charge-discharge curves. **b** areal capacitance and **c** rate stability of the fabricated MSCs without anneal

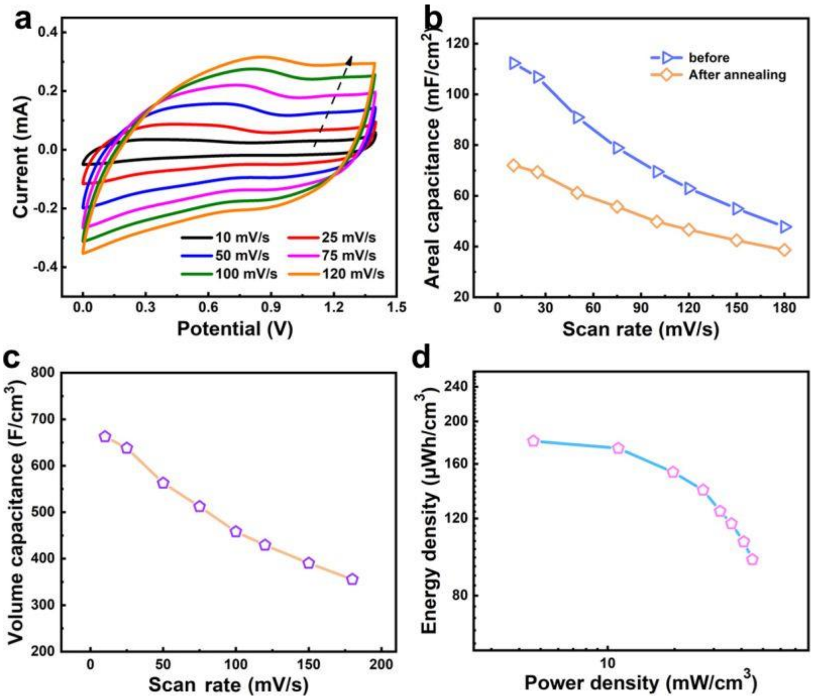

Fig. S3 a CV curves, **b** compared areal capacitance, **c** volume capacitance and **d** range plot of *in-situ* annealed Zn-ion MSCs

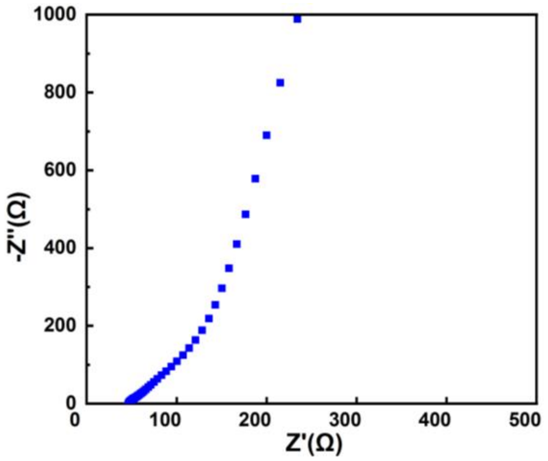

Fig. S4 Nyquist impedance of the $Ti_3C_2T_x$ based concentric circular Zn-ion MSC after annealing treatment

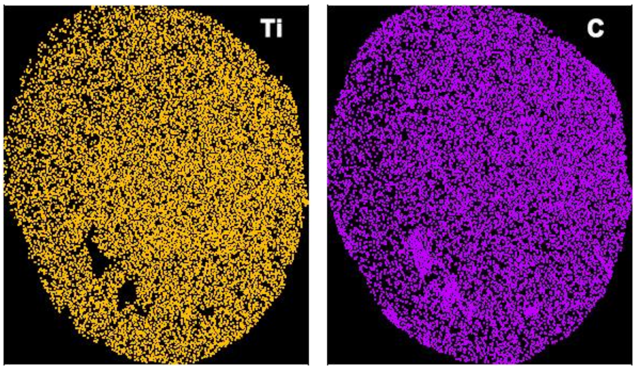

Fig. S5 Ti and C element dispersion in the $Ti_3C_2T_x$ cathode, corresponding to Fig. 4b

Caculation:

Areal and volumetric capacitances can be calculated by the equations:

$$C_A = \frac{\int_0^v I \, dv}{SVA}$$

Where C_A represents the specific areal/volume capacitance, *I* is the current, *S* stands for the scan rate, *V* is the potential in the CV curve, *A* is the areal or volume of the devices.

The energy density and power density:

$$E = C_A \times \Delta V^2 / 7200$$

$$P = E \times 3600 / \Delta t$$

Where *E* represents the energy density, *P* is the power density, and Δt is the total discharge time.
